# Supplementary material for: Citrullination of glucokinase is linked to autoimmune diabetes
Source: Nat Commun. 2022 Apr 6;13:1870. doi: 10.1038/s41467-022-29512-0 (PMC8986778; doi:10.1038/s41467-022-29512-0)
Supplement: Supplementary file 1 — Supplementary Information [file 41467_2022_29512_MOESM1_ESM.pdf]

Citrullination of glucokinase is linked to autoimmune diabetes

Supplementary Tables and Figures

Supplementary Table 1: Correlation analysis of anti-GK and anti-citrullinated-GK with other known T1D autoantibodies.

Pearson r	anti-GK			anti-citrullinated-GK		
	r	p (two-tailed)	95%CI	r	p (two-tailed)	95%CI
anti-insulin	0.07407	0.591	-0.1951 to 0.3328	0.1358	0.323	-0.1344 to 0.3871
anti-GAD65	-0.0185	0.9374	-0.2754 to 0.2552	0.04039	0.7697	-0.2273 to 0.3024
anti-IA2	0.384	0.0038*	0.1322 to 0.5893	0.2823	0.0368*	0.01835 to 0.5094
anti-ZnT8	-0.2077	0.1281	-0.4483 to 0.06096	-0.196	0.1516	-0.4385 to 0.07315

n=55; 95%CI, 95% confidence interval

Supplementary Table 2. Binding of Predicted Glucokinase Peptides to DR04:01

Peptide	Sequence	IC_{50} (μM)[a,b]
GK 17-31	EQILAEFQLQEEDLK	>50
GK 44-58	GL[Cit]LETHEEASVKML	>50
GK 54-68	SVKMLPTYV[Cit]STPEG	>50
**GK 58-72**	**LPTYV[Cit]STPEGSEVG**	**30.9**
GK 71-85	VGDFLSLDLGGTNFR	>50
**GK 81-95**	**GTNFRVMLVKVGEGE**	**15.3**
GK 83-98	NFRVMLVKVGEGEEG	>50
**GK 120-134**	**EMLFDYISECISDFL**	**40.1**
GK 145-159	PLGFTFSFPVRHEDI	>50
GK 168-182	TKGFKASGAEGNNVV	>50
**GK 192-206[c]**	**RGDFEMDVVAMVNDT**	**0.35**
**GK 199-214[c]**	**VVAMVNDTVATMISCY**	**2.5**
GK 222-236	VGMIVGTGCNACYME	>50
**GK 231-245**	**NACYMEEMQNVELVE**	**0.03**
GK 240-254	NVELVEGDEGRMCVN	>50
GK 248-262	EGRMCVNTEWGAFGD	>50
GK 250-264	RMCVNTEWGAFGDSG	>50
GK 254-268	NTEWGAFGDSGELDE	>50
GK 257-271	WGAFGDSGELDEFLL	>50
**GK 266-280[c]**	**LDEFLLEYDRLVDES**	**5.6**
**GK 270-284[c]**	**LLEYDRLVDESSANP**	**2.0**
GK 274-288	DRLVDESSANPGQQL	>50
GK 286-300	QQLYEKLIGGKYMGE	>50
**GK 294-308**	**GGKYMGELV[Cit]LVLLR**	**4.3**
GK 306-320	LLRLVDENLLFHGEA	>50
GK 311-324	DENLLFHGEASEQL	>50
GK 313-327	NLLFHGEASEQL[Cit]TR	>50
GK 327-341	RGAFET[Cit]FVSQVESD	>50
GK 331-344	ET[Cit]FVSQVESDTGD	>50
**GK 346-360[c]**	**KQIYNILSTLGL[Cit]PS**	**0.47**
GK 349-363	YNILSTLGL[Cit]PSTTD	>50
GK 352-366	LSTLGL[Cit]PSTTDCDI	>50
GK 365-378	DIV[Cit]RACESVST[Cit]A	>50
GK 387-401	AGVIN[Cit]M[Cit]ES[Cit]SEDV	>50
GK 398-412	SEDVM[Cit]ITVGVDGSV	>50
GK 410-424	GSVYKLHPSFKE[Cit]FH	>50
GK 416-439	HPSFKE[Cit]FHASVR[Cit]L	>50
GK 435-449	EITFIESEEGSG[Cit]GA	>50

[a] IC_{50} represents the peptide concentration that displaces half of the reference peptide.
[b] Peptides selected for *in vitro* studies (based on IC_{50} values) are shown in boldface.
[c] Peptides subsequently shown to be immunogenic.

Supplementary Table 3. Effect of Citrulline or Arg Substitution

Peptide	Amino acid sequence[a,b]	IC_{50} (μM)[c,d]
GK 266 WT	LDEFL**LEYDRLVDE**S	5.6
GK 266 Cit	LDEFL**LEYDXLVDE**S	9.5
GK 270 WT	LLE**YDRLVDESS**ANP	2.0
GK 270 Cit	LLE**YDXLVDESS**ANP	8.6
GK 346 WT	KQI**YNILSTLGL**RPS	0.47
GK 346 Cit	KQI**YNILSTLGL**XPS	0.54

[a] The most likely motif is bolded in each sequence.
[b] X indicates a citrulline residue.
[c] IC50 represents the peptide concentration that displaces half of the reference peptide - representative values from two replicate experiments are shown.
[d] Peptides with no detectable binding were assigned an IC50 >50 μM (limit of detection).

Supplementary Table 4. DRB1*4:01+ subjects with diabetes

Subject ID	Age[a]	Sex	HLA-DR Type[b]	Time Since Diagnosis[a]
T1D #1	45	Female	*04:01/*04-	10.6 years
T1D #2	31	Male	*04:01/*04:04	25.0 years
T1D #3	34	Male	*04:01/*03-	31.0 years
T1D #4	45	Male	*04:01/ND	37.0 years
T1D #5	30	Female	*04:01/*07:01	7.0 years
T1D #6	43	Female	*04:01/*13-	40.0 years
T1D #7	54	Male	*04:01/*03:01	5.8 years
T1D #8	57	Female	*04:01/*03:01	26.6 years
T1D #9	43	Female	*04:01/*03-	11.0 years
T1D #10	32	Female	*04:01/*01:01	11.8 years

[a] The mean age of patients was 41.7 years. The average time since diagnosis was 20.6 years.
[b] ND indicates that the DR type could not be determined by the method used, dash mark (-) indicates that the high resolution DR type was not conclusive.

Supplementary Table 5. DRB1*04:01+ healthy subjects

Subject ID	Age[a]	Sex	HLA-DR Type[b]
Control #1	47	Male	*04:01/*11:01
Control #2	47	Female	*04:01/*01:01
Control #3	58	Female	*04:01/*07:01
Control #4	44	Male	*04:01/*07:01
Control #5	30	Female	*04:01/*01:02
Control #6	35	Female	*04:01/*11-
Control #7	31	Male	*04:01/*03-
Control #8	58	Male	*04:01/*13:02
Control #9	65	Female	*04:01/*07:01
Control #10	36	Female	*04:01/*07:01

[a] The mean age of controls was 45.1 years. Healthy subjects under the age of 18 could not be recruited.
[b] Dash mark (-) indicates that the high resolution DR type was not conclusive.

Supplementary Table 6. Citrullinated peptides of PAD-treated rhGK identified by mass spectrometry

Modified Arginine	Peptide sequence[a]
R46	GL**X**LETHEEASVK
R186	ASGAEGNNVVGLL**X**DAIK
R327	T**X**GAFETR
R333	GAFET**X**FVSQVESDTGDR; GAFET**X**FVSQVESDTGDRK
R345	GAFET**X**FVSQVESDTGD**X**K
R358	QIYNILSTLGL**X**PSTTDCDIVR
R369	**X**ACESVSTR; **X**ACESVST**X**AAHMCSAGLAGVINR
R377	**X**ACESVST**X**AAHMCSAGLAGVINR; ACESVST**X**AAHMCSAGLAGVINR
R392	AAHMCSAGLAGVIN**X**MR
R403	SEDVM**X;** SEDVM**X**ITVGVDGSVYK
R422	E**X**FHASVR

[a] **X** = citrulline

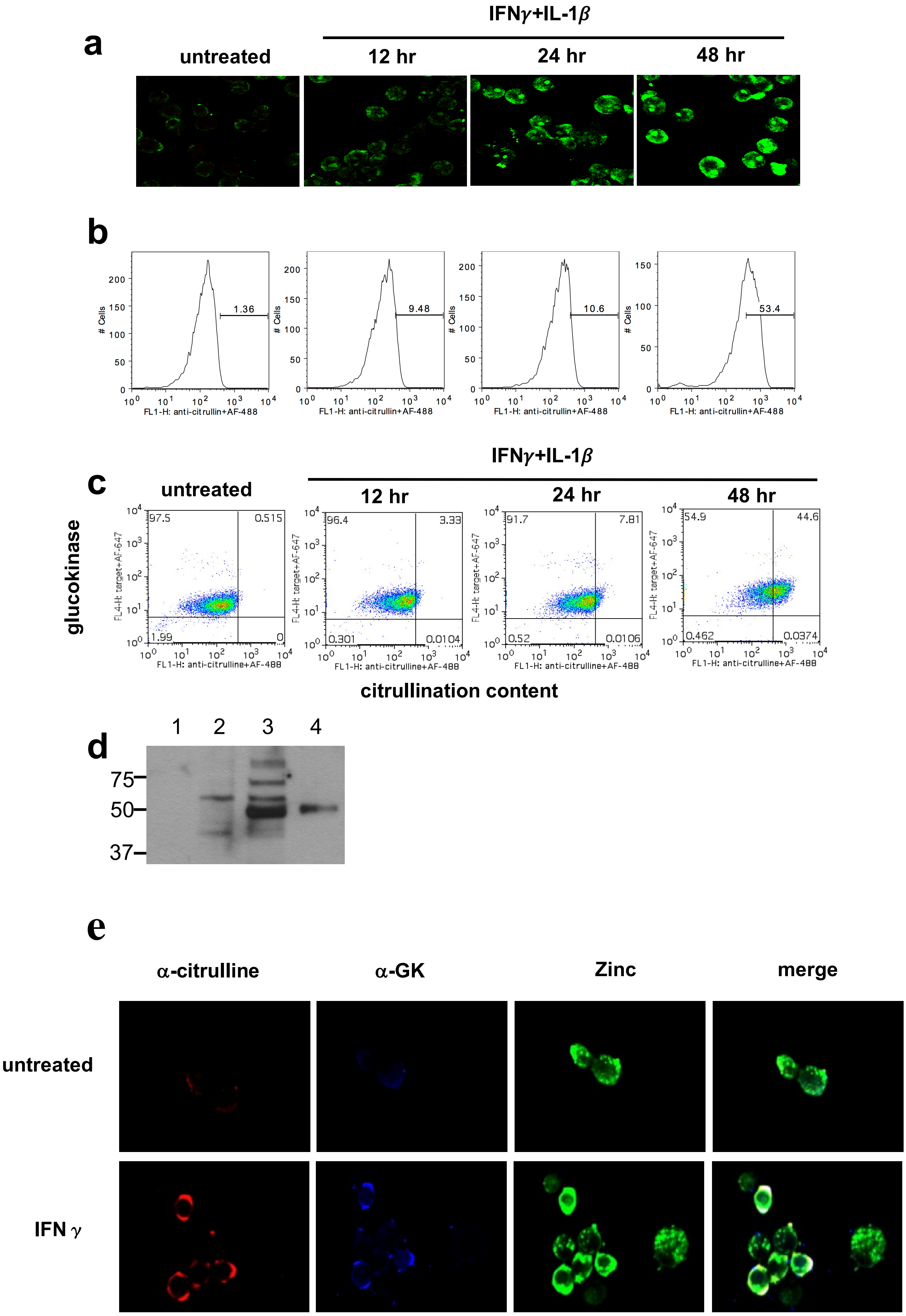
IFNγ+IL-1β
a
untreated
12 hr
24 hr
48 hr
b
Cells
1.36
9.48
10.6
53.4
FL1-H: anti-citrullin+AF-488
IFNγ+IL-1β
c
untreated
12 hr
24 hr
48 hr
glucokinase
FL4-H: target+AF-647
97.5
0.515
1.99
0
96.4
3.33
0.301
0.0104
91.7
7.81
0.52
0.0106
54.9
44.6
0.462
0.0374
FL1-H: anti-citrulline+AF-488
citrullination content
d
1
2
3
4
75
50
37
e
α-citrulline
α-GK
Zinc
merge
untreated
IFN γ

Supplementary Figure 1. Glucokinase citrullination in beta cells under inflammatory stress. a-c Representative confocal images (**a**) and FACS plots (**b, c**) showing intracellular protein citrullination among control and IFNγ (1000U/ml) plus IL-1β (50U/ml) treatment at different time point (between 12 and 48 hrs) in INS-1 beta cells. **d** The INS-1 cell lysate was immunoprecipitated using anti-glucokinase (lane 2 and 3) and isotype control rabbit IgG (lane 1) and immunoblotted with anti-peptidyl citrulline. Lane 1 and 2: untreated INS-1 cell lysate. Lane 3: IFNγ and IL-1β treated- INS-1 cell lysate. Lane 4: PAD-treated recombinant human glucokinase (PAD-rhGK; GK indicated by arrow). **e** Representative fluorescence images of sorted human beta cells with or without IFNγ (100ng/ml) treatment with triple immunofluorescence for citrullination (red), glucokinase (blue), and Zinc (green).

a

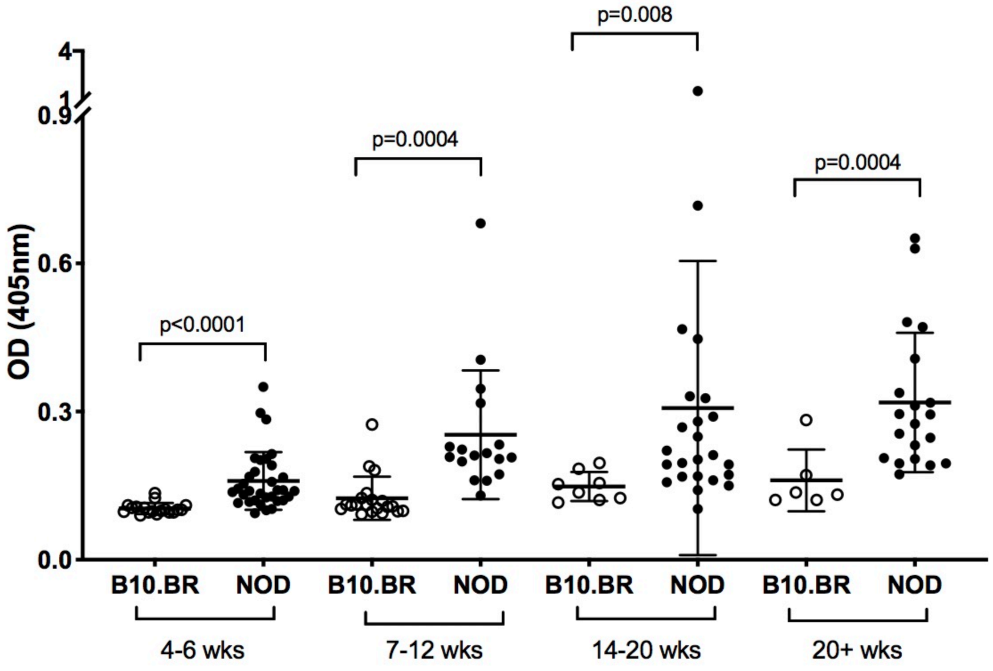
p=0.008
p=0.0004
p=0.0004
p<0.0001
4
1
0.9
0.6
0.3
0.0
OD (405nm)
B10.BR
NOD
B10.BR
NOD
B10.BR
NOD
B10.BR
NOD
4-6 wks
7-12 wks
14-20 wks
20+ wks

b

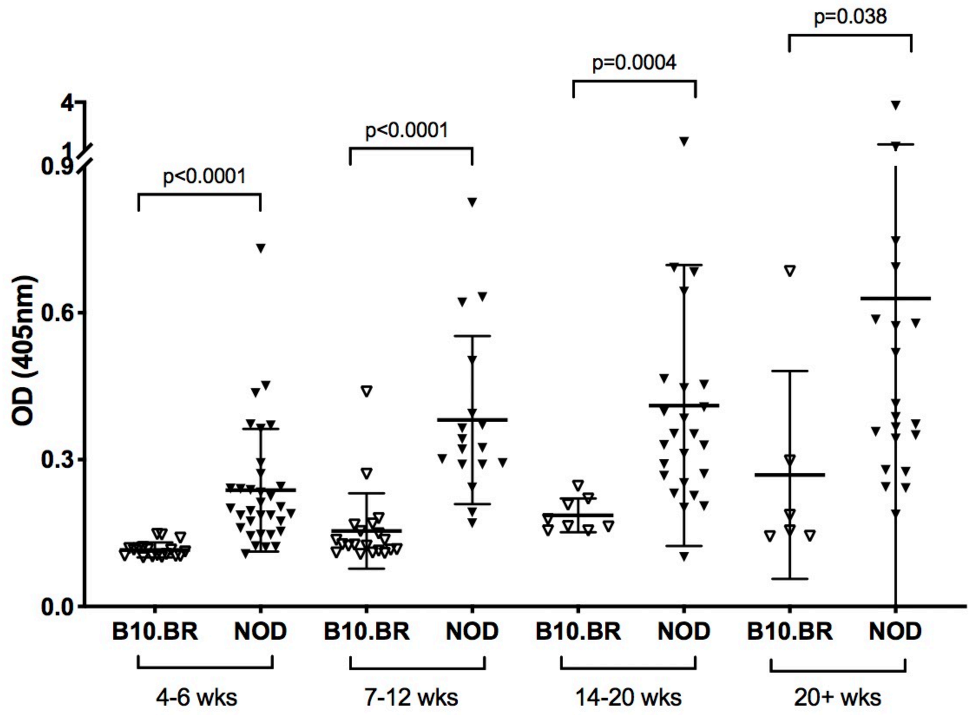
p=0.038
p=0.0004
p<0.0001
p<0.0001
4
1
0.9
0.6
0.3
0.0
OD (405nm)
B10.BR
NOD
B10.BR
NOD
B10.BR
NOD
B10.BR
NOD
4-6 wks
7-12 wks
14-20 wks
20+ wks

c

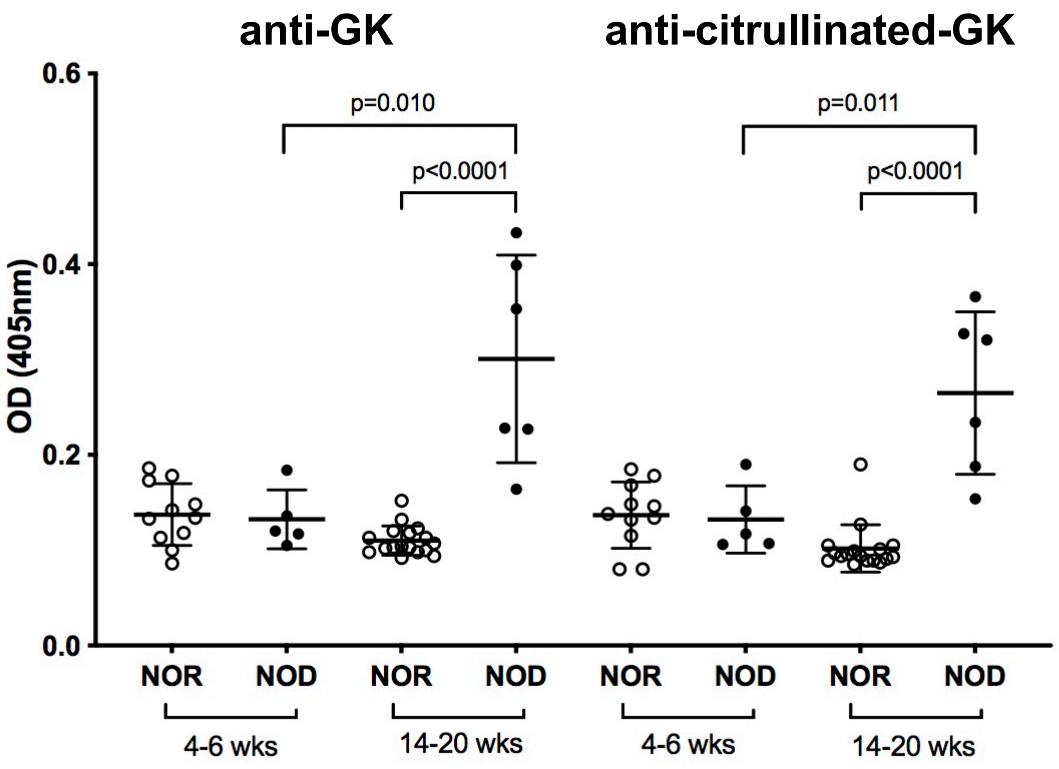

Supplementary Figure 2. The autoantibodies against glucokinase and citrullinated glucokinase were subgrouped by different ages from B10.BR, NOR and NOD mice. a-b The serum levels of anti-GK (**a and c**) and anti-citrullinated GK (**b and c**) from NOD mice (a-c), B10.BR (a and b) and NOR (c) mice were measured by ELISA as described in Methods. The error bars indicated SD of mean. Statistical analysis was performed using Student's *t*-test.

1 mlddrarmea akkekveqil aefqlqeedl kkvmrrmqke mdrgl**R**leth eeasvkmlpt

61 yvrstpegse vgdflsldlg gtnfrvmlvk vgegeegqws vktkhqmysi pedamtgtae

121 mlfdyiseci sdfldkhqmk hkklplgftf sfpvrhedid kgillnwtkg fkasgaegnn

181 vvgll**R**daik rrgdfemdvv amvndtvatm iscyyedhqc evgmivgtgc nacymeemqn

241 velvegdegr mcvntewgaf gdsgeldefl leydrlvdes sanpgqqlye kliggkymge

301 lvrlvllrlv denllfhgea seqlrt**R**gaf et**R**fvsqves dtgd**R**kqiyn ilstlgl**R**ps

361 ttdcdivr**R**a cesvst**R**aah mcsaglagvi n**R**mresrsed vm**R**itvgvdg svyklhpsfk

421 e**R**fhasvrrl tpsceitfie seegsgrgaa lvsavackka cmlgq

Supplementary Figure 3. Sequence of human glucokinase demonstrated 11 arginines targeted by *in vitro* citrullination (shown as bold capital "R"). The five immunogenic peptides identified from tetramer staining assays are shown as underlined. *; Arg 358.

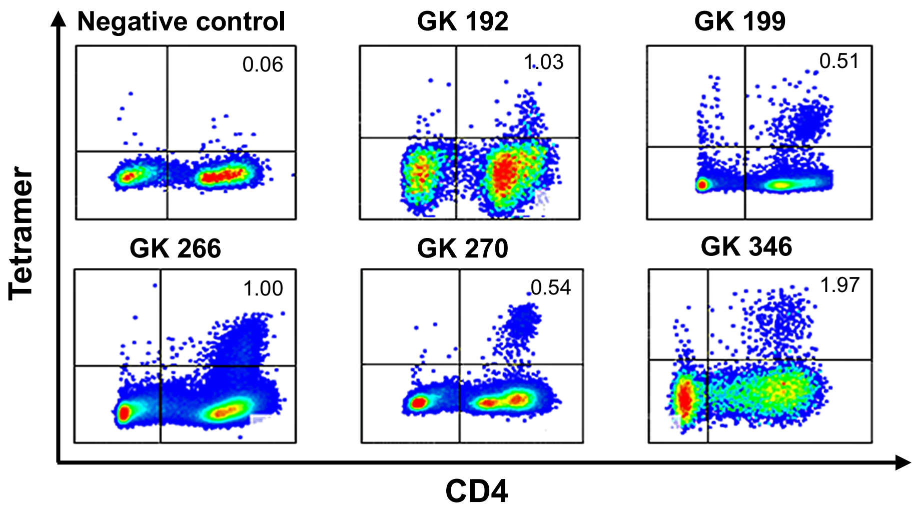

Supplementary Figure 4. Assessing the *in vitro* immunogenicity of glucokinase peptides. PBMC from subjects with type 1 diabetes (T1D) with DR0401 haplotypes were expanded by stimulating for two weeks with groups of GK peptides and then stained with corresponding peptide loaded tetramers. The staining panels above show the representative positive (GK 192, GK 199, GK 266, GK 270, and GK 346) and a negative control (unloaded) tetramer staining result, displayed as CD4 versus tetramer after gating for CD3 lymphocytes.

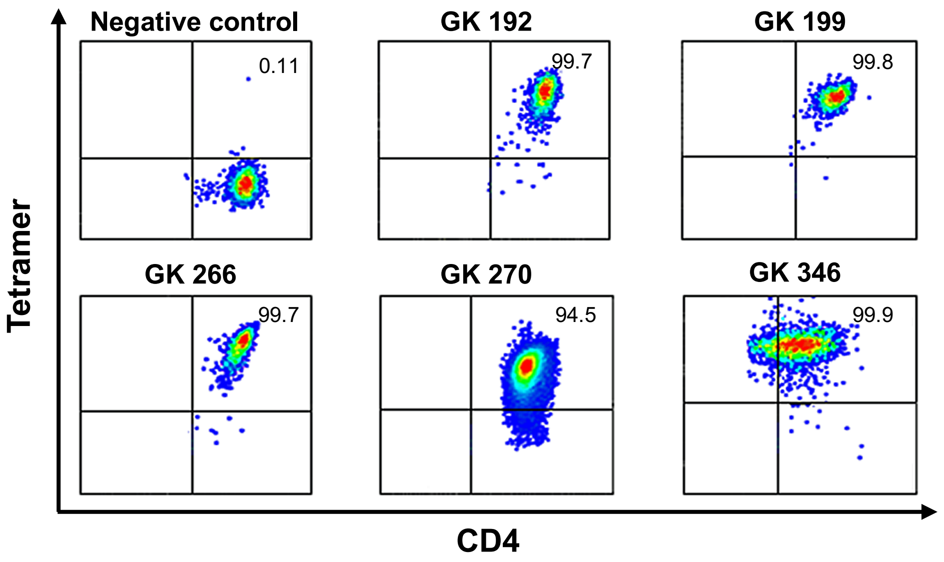

Supplementary Figure 5. Tetramer staining of glucokinase reactive T cell clones. T cell clones representing five glucokinase specificities were sorted, expanded, and re-stained with peptide loaded DR0401 tetramers. Each the corresponding clone was stained by the corresponding glucokinase tetramer and negative control, further supporting the specificity of the tetramer staining observed in the *in vitro* experiments.

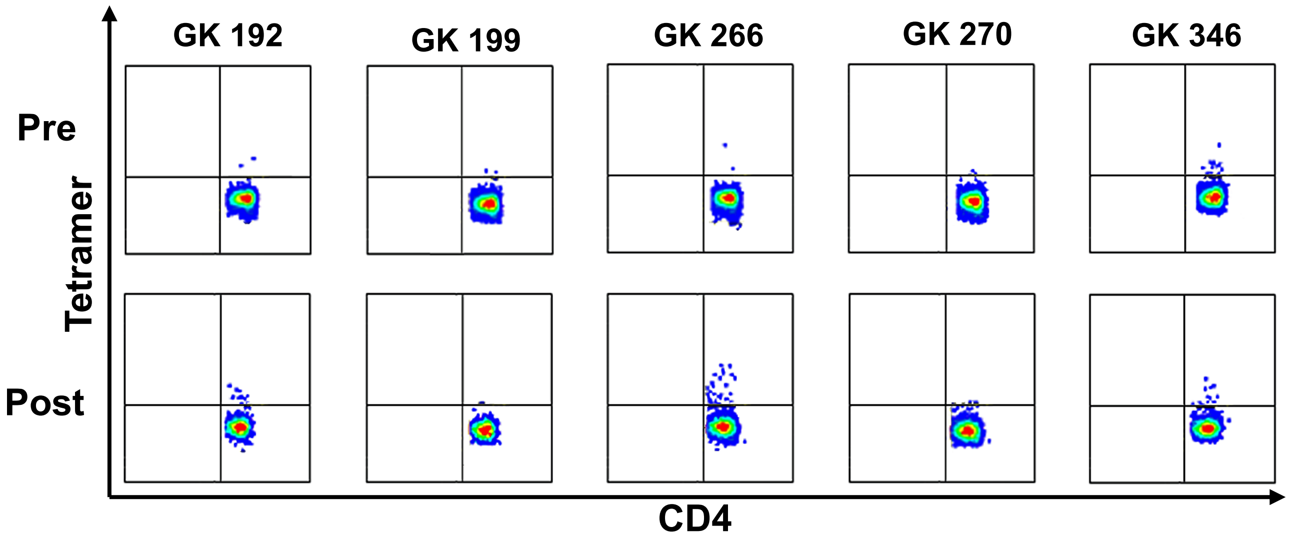

Supplementary Figure 6. Glucokinase specific CD4$^+$ T cells can be directly detected in subjects with T1D. For each subject, tetramer staining was performed with PE, PE-Cy5, and/or PE-CF594 labeled tetramers to enumerate CD4 T cells for each of the five individual GK specificities using two parallel staining tubes for each subject. The panels above show tetramer staining results from a representative subject. Each upper panel shows the pre-column fraction, used to determine the total number of CD4+ T cells in the unmanipulated sample and to set a threshold for positive tetramer staining for the indicated GK epitope. Each lower panel shows the corresponding enriched fraction, used to determine the total number of epitope-specific CD4 T cells in the sample. Cells were gated based on size, viability and lack of CD14/CD19 expression, and is displayed as a plot of CD4 versus tetramer (PE, PE-Cy5, or PE-CF594 labeled) as shown.

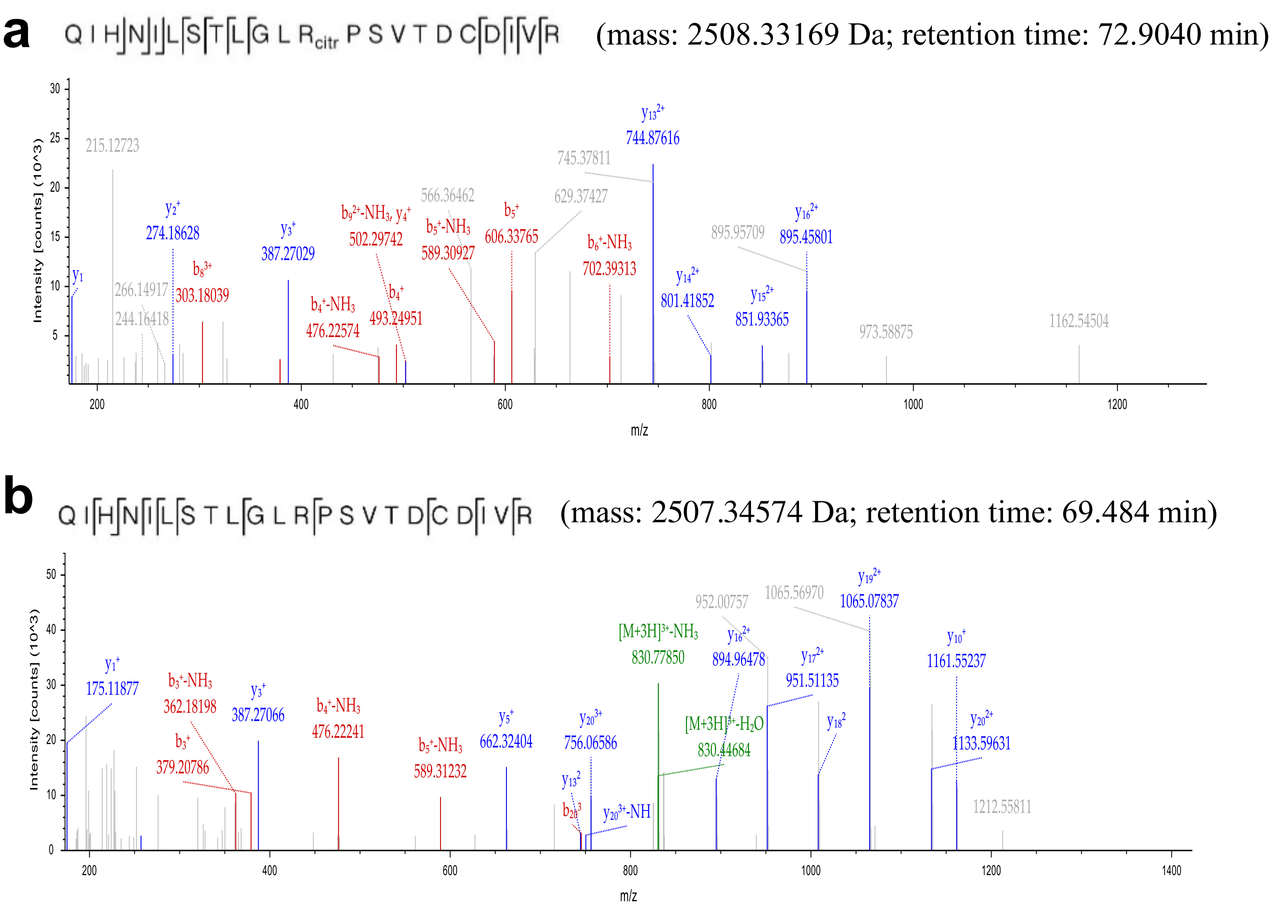

Supplementary Figure 7. Citrullination identification in glucokinase protein. INS-1E cells were cultured with IFNγ (500 units/mL) and IL-1β (10 units/mL) for 16 hrs. Cell lysates were then immunoprecipitated using anti-GK and subjected to mass spectrometry analysis as described in Methods. Arg^{358} residue of glucokinase was identified to be citrullinated in (a) cytokine treated- but not in (b) untreated INS-1E beta cells. The mass and retention time of the peptide are indicated with the peptide sequence above the individual MS/MS spectra.
