# Supplementary material for: Citrullination of glucokinase is linked to autoimmune diabetes
Source: Nat Commun. 2022 Apr 6;13:1870. doi: 10.1038/s41467-022-29512-0 (PMC8986778; doi:10.1038/s41467-022-29512-0)
Supplement: Supplementary file 3 — Reporting Summary [file 41467_2022_29512_MOESM3_ESM.pdf]

nature portfolio

Corresponding author(s): Mark J. Mamula

Last updated by author(s): Feb 28, 2022

Reporting Summary

Nature Portfolio wishes to improve the reproducibility of the work that we publish. This form provides structure for consistency and transparency in reporting. For further information on Nature Portfolio policies, see our Editorial Policies and the Editorial Policy Checklist.

Statistics

For all statistical analyses, confirm that the following items are present in the figure legend, table legend, main text, or Methods section.

n/a	Confirmed	
☐	☒	The exact sample size (n) for each experimental group/condition, given as a discrete number and unit of measurement
☐	☒	A statement on whether measurements were taken from distinct samples or whether the same sample was measured repeatedly
☐	☒	The statistical test(s) used AND whether they are one- or two-sided<br>*Only common tests should be described solely by name; describe more complex techniques in the Methods section.*
☒	☐	A description of all covariates tested
☒	☐	A description of any assumptions or corrections, such as tests of normality and adjustment for multiple comparisons
☐	☒	A full description of the statistical parameters including central tendency (e.g. means) or other basic estimates (e.g. regression coefficient) AND variation (e.g. standard deviation) or associated estimates of uncertainty (e.g. confidence intervals)
☐	☒	For null hypothesis testing, the test statistic (e.g. F, t, r) with confidence intervals, effect sizes, degrees of freedom and P value noted<br>*Give P values as exact values whenever suitable.*
☒	☐	For Bayesian analysis, information on the choice of priors and Markov chain Monte Carlo settings
☒	☐	For hierarchical and complex designs, identification of the appropriate level for tests and full reporting of outcomes
☐	☒	Estimates of effect sizes (e.g. Cohen's d, Pearson's r), indicating how they were calculated

Our web collection on statistics for biologists contains articles on many of the points above.

Software and code

Policy information about availability of computer code

Data collection	Leica SP5 II laser scanning confocal microscope, Synergy HT Multi-Mode Reader, FACSCanto, FACSCalibur, FACS Aria, FACS AriaII, FACS LSRII, FACSDiva (v. 8.0), Q Exactive Orbitrap mass spectrometer (ThermoScientific) coupled to an Ultimate 3000 UPLC system (Dionex, Thermo Scientific) equipped with an Acclaim PepMap100 pre-column (C18, particle size 3 µm, pore size 100 Å, diameter 75 µm, length 20 mm, Thermo Scientific) and a C18 PepMap RSLC (particle size 2 µm, pore size 100 Å, diameter 50 µm, length 150 mm, Thermo Scientific) using a linear gradient (300 nL/min), and QE-Plus mass spectrometer coupled to a NanoACQUITY UPLC.
Data analysis	Graphpad Prism v. 9.0, Microsoft excel 2018, Scaffold Q+S 4.11.0, Scaffold PTM 3.3.0, Proteome Discoverer Software v. 2.2, MASCOT Search Engine v. 2.7, FlowJo v. 9.9 and v. 10.7.1

For manuscripts utilizing custom algorithms or software that are central to the research but not yet described in published literature, software must be made available to editors and reviewers. We strongly encourage code deposition in a community repository (e.g. GitHub). See the Nature Portfolio guidelines for submitting code & software for further information.

Data

Policy information about availability of data

All manuscripts must include a data availability statement. This statement should provide the following information, where applicable:

- Accession codes, unique identifiers, or web links for publicly available datasets
- A description of any restrictions on data availability
- For clinical datasets or third party data, please ensure that the statement adheres to our policy

The mass spectrometry proteomics data have been deposited to the ProteomeXchange Consortium via the PRIDE partner repository with the dataset identifier PXD028825. Source data are provided with this paper for the following figures: Fig 2b, 2c, Fig 3a-3e, Fig 4b, Fig 5c, 5d, Supplementary Table 1, Supplementary Fig

2a-2c in the separate spreadsheets of "Source Data-File 1" file. The representative MS/MS spectra with annotation of the neutral loss of isocyanic acid for all citrullinated sites identified in PAD-treated rhGK are listed in the separate spreadsheets of "Source Data-File 2" file.

Field-specific reporting

Please select the one below that is the best fit for your research. If you are not sure, read the appropriate sections before making your selection.

- [x] Life sciences
- [ ] Behavioural & social sciences
- [ ] Ecological, evolutionary & environmental sciences

For a reference copy of the document with all sections, see nature.com/documents/nr-reporting-summary-flat.pdf

Life sciences study design

All studies must disclose on these points even when the disclosure is negative.

Sample size	N= minimum of 3 biological replicates and between 3 and 6 technical replicates were used to generate data. Sample size was designed on the basis of trial experiments or the results from the first iteration of each experiment and were increased to achieve statistical significance if necessary.
Data exclusions	No data was excluded.
Replication	All attempts at 3 or more replication were successfully reproducible.
Randomization	Randomization was not applicable in this study. Sex and age matched animals were used for experimental and control groups. We chose patient groups and/or samples with confirmed diagnostic criteria and clinical criteria for type 1 diabetes or from normal healthy individuals.
Blinding	1) For murine subjects, blinding is not relevant since the genetics were identical for all processed murine subjects in the same experiment.<br>2) For human subjects, all transportation and experiments were done by investigators blinded to the group allocation.

Reporting for specific materials, systems and methods

We require information from authors about some types of materials, experimental systems and methods used in many studies. Here, indicate whether each material, system or method listed is relevant to your study. If you are not sure if a list item applies to your research, read the appropriate section before selecting a response.

Materials & experimental systems

n/a	Involved in the study	
☐	☒	Antibodies
☐	☒	Eukaryotic cell lines
☒	☐	Palaeontology and archaeology
☐	☒	Animals and other organisms
☐	☒	Human research participants
☒	☐	Clinical data
☒	☐	Dual use research of concern

Methods

n/a	Involved in the study	
☒	☐	ChIP-seq
☐	☒	Flow cytometry
☒	☐	MRI-based neuroimaging

Antibodies

Antibodies used

anti-glucokinase (Proteintech, 19666-1-AP, Lot # 00014819 and # 00023801)
anti-peptidyl-citrulline (Millipore, MABN328, clone F95, Lot # 3130480)
anti-citrulline antibody (Abcam, ab6464)
Goat anti-Human IgG-AP, (SouthernBiotech, 2040-04)
Goat anti-Mouse IgM (u chain specific) Horseradish Peroxidase conjugated (Sigma-Aldrich, AP128P)
Goat anti-rabbit IgG (H+L) Alexa Fluor 647 conjugated (ThermoFisher, A21244)
Goat anti Mouse IgM Alexa Fluor 488 conjugated (ThermoFisher, A21042)
anti-CD4 BV650 conjugated (Biolegend, 317436, clone RPA-T4, Lot # B284308)
anti-CD3 APC conjugated (eBiosciences, 17-0038-42, clone UCHT1, Lot # 2071259)
anti-CD25 FITC conjugated (Biolegend, 302604, clone BC96, Lot # B253407)
anti-CD4 V500 conjugated (BD Bioscience, 560768, clone RPA-T4, Lot # 8325744)
anti-CD14 PerCP-Cy5.5 conjugated (eBiosciences, 45-0149-42, clone 61D3, Lot # 1933247)
anti-CD19 PerCP-Cy5.5 conjugated (eBiosciences, 45-0198-42, clone SJ25C1, Lot # 4297194)
anti-CD45RA AF700 conjugated (BD Bioscience, 560673, clone HI100, Lot # 7047628)
anti-CXCR3 FITC conjugated (Biolegend, 353704, clone G025H7, Lot # B185804)
anti-CCR6 BV421 conjugated (Biolegend, 353408,clone G034E3, Lot # B206002)

	anti-CCR4 BV605 conjugated (Biolegend, 359418, clone L291H4, Lot # B265240)<br>ViaProbe PerCP-Cy5.5 conjugated (BD Bioscience, 555815, Lot # 7146688)
Validation	All commercially available antibodies used are validated. Please check manufacturer's link for validation of antibodies.<br><br>https://www.ptglab.com/products/GCK-Antibody-15629-1-AP.htm<br>https://www.ptglab.com/products/GCK-Antibody-19666-1-AP.htm<br>https://www.emdmillipore.com/US/en/product/Anti-peptidyl-citrulline-clone-F95-Antibody,MM_NF-MABN328?ReferrerURL=https%3A%2F%2Fwww.google.com%2F#documentation<br>https://www.abcam.com/citrulline-antibody-ab6464.html?#description_references<br>https://www.southernbiotech.com/?catno=2040-04&type=Polyclonal#&panel1-2&panel2-1<br>https://www.sigmaaldrich.com/US/en/product/mm/ap128p<br>https://www.thermofisher.com/antibody/product/Goat-anti-Rabbit-IgG-H-L-Cross-Adsorbed-Secondary-Antibody-Polyclonal/A-21244<br>https://www.thermofisher.com/antibody/product/Goat-anti-Mouse-IgM-Heavy-chain-Cross-Adsorbed-Secondary-Antibody-Polyclonal/A-21042<br>https://www.biolegend.com/en-us/products/brilliant-violet-650-anti-human-cd4-antibody-7786<br>https://www.thermofisher.com/antibody/product/CD3-Antibody-clone-UCHT1-Monoclonal/17-0038-42<br>https://www.biolegend.com/en-us/products/fitc-anti-human-cd25-antibody-615<br>https://www.bdbiosciences.com/en-us/products/reagents/flow-cytometry-reagents/research-reagents/single-color-antibodies-ruo/v500-mouse-anti-human-cd4.560768<br>https://www.thermofisher.com/antibody/product/CD14-Antibody-clone-61D3-Monoclonal/45-0149-42<br>https://www.thermofisher.com/antibody/product/CD19-Antibody-clone-SJ25C1-Monoclonal/45-0198-42<br>https://www.bdbiosciences.com/en-us/products/reagents/flow-cytometry-reagents/research-reagents/single-color-antibodies-ruo/alexa-fluor-700-mouse-anti-human-cd45ra.560673<br>https://www.biolegend.com/en-us/products/fitc-anti-human-cd183-cxcr3-antibody-7578<br>https://www.biolegend.com/en-us/search-results/brilliant-violet-421-anti-human-cd196-ccr6-antibody-7547<br>https://www.biolegend.com/en-us/products/brilliant-violet-605-anti-human-cd194-ccr4-antibody-8854<br>https://www.bdbiosciences.com/en-us/products/reagents/flow-cytometry-reagents/research-reagents/single-color-antibodies-ruo/cell-viability-solution.555815

Eukaryotic cell lines

Policy information about cell lines

Cell line source(s)	INS-1 cell line was from the supplier, ATCC. INS-1E cell line was kindly provided from Prof. C. Wollheim, Centre Medical Universitaire, Geneva, Switzerland.
Authentication	INS-1 cells were karyotyped and confirmed for phenotype in our laboratory. INS-1E cell lines was karyotyped by the contributor and phenotype confirmed on a regular basis by evaluating glucose induced-insulin secretion.
Mycoplasma contamination	Absence of Mycoplasma contamination was confirmed by PCR analysis using Venor®GeM Mycoplasma PCR Detection Kit (Minerva Biolabs, Berlin, Germany).
Commonly misidentified lines<br>(See ICLAC register)	none

Animals and other organisms

Policy information about studies involving animals; ARRIVE guidelines recommended for reporting animal research

Laboratory animals	Female NOD/ShiLt, NOR/LtJ, C57BL/6, and B10.BR mice, 4-30w.
Wild animals	No wild animals were used.
Field-collected samples	No field-collected samples were used.
Ethics oversight	All animal studies were performed in accordance with the guidelines of the Yale University Institutional Animal Care and Use Committee.

Note that full information on the approval of the study protocol must also be provided in the manuscript.

Human research participants

Policy information about studies involving human research participants

Population characteristics	The relevant population characteristics are available within the "Methods" and/or its "supplementary information", supplementary Table 4 and 5.
Recruitment	For serum and peripheral blood samples: Subjects for this study are recruited through the BRI Diabetes and Control Registry modules of the Immune Mediated Disease Registry and Repository. New potential participants may contact BRI unsolicited through the BRI Diabetes Research Line (1-800-888-4187), BRI website, or email (diabetes@benaroyaresearch.org). Subjects

	are also recruited via physician referral, through community diabetes events (i.e. camps, ADA/JDRF events and walks, and patient forums), letters and advertisements. They may also be recruited through collaborating institutions with appropriate IRB oversight. We have not identified any specific biases in our population based on this broad recruitment strategy, but any such biases that may exist are unlikely to impact this cross-sectional retrospective study.
Ethics oversight	For serum and peripheral blood samples: This study was reviewed and approved by the Benaroya Research Institute Institutional Review Board, with annual continuing review.<br><br>For human islets: This work is designated as "Not human subjects research" by the Yale Institutional Review Board since it did not involve data obtained through intervention or interaction with the individual and did not contain identifiable private information (per 45 CFR 46.102)

Note that full information on the approval of the study protocol must also be provided in the manuscript.

Flow Cytometry

Plots

Confirm that:

- [x] The axis labels state the marker and fluorochrome used (e.g. CD4-FITC).
- [x] The axis scales are clearly visible. Include numbers along axes only for bottom left plot of group (a 'group' is an analysis of identical markers).
- [x] All plots are contour plots with outliers or pseudocolor plots.
- [x] A numerical value for number of cells or percentage (with statistics) is provided.

Methodology

Sample preparation	For INS-1 cells:<br>Cells were seeded in 6-well plates (1,000,000 cells per condition) cultured with or without recombinant mouse IFNγ (1000 units/mL; R&D Systems) and recombinant human IL-1β (50 units/mL; R&D Systems) for 48 hours. After trypsinization, cells were fixed by 95% methanol and 4% paraformaldehyde for 30 min on ice. Cells were stained with anti-peptidyl-citrulline, clone F95 (Millipore), and anti-mouse Alexa Fluor 488 (Life Technology) for intracellular citrullination level and/or with anti-glucokinase (Proteintech) and anti-rabbit Alexa Fluor 647 (Life Technology) for glucokinase expression in staining buffer (PBS containing 1% BSA and 0.1% Tween-20).<br><br>For human T cells:<br>The sample preparation for each experiment setting are available within the "Methods" and its "supplementary information", supplementary Figure 3, 4 and 5.<br><br>For human beta cells:<br>Single-cell suspensions were prepared from pancreatic islets by trypsin dissociation and then stained with FluoZin-3 (Invitrogen) and TMRE (Life Technologies) for cell sorting.
Instrument	For INS-1 cells: FACSCalibur<br>For human T cells: FACS Aria, FACSCanto and FACS LSRII<br>For human islet beta cells: FACS AriaII
Software	For INS-1 cells: FlowJo v. 9.9<br>For human T cells: FlowJo v. 10.7.1<br>For human islet beta cells: FACSDiva v. 8.0 and FlowJo v. 9.9
Cell population abundance	For INS-1 cells:<br>At least 250,000 cells were used per sample for intracellular staining. For each run, at least 10,000 events were analyzed.<br><br>For human T cells:<br>Each experiment setting are available within the "Methods" and its "supplementary information", supplementary Figure 3, 4 and 5. Each pre & post tube was run to completion (i.e. virtually no FACS buffer remained).<br><br>For human beta cells, TMRE+ and zinc+ was used for sorting with a FACSAria II and the percentage was around 30-60% of total pancreatic islet cells.
Gating strategy	For INS-1 cells:<br>Cell were first gated by FSC/SSC to exclude dead cells and debris and then positive and negative threshold were set using an isotype Ig control with the same fluorophore.<br><br>For human T cells:<br>Cells were first gated on FSC-A/SSC-A to determine bulk lymphoctye population. Second, cells were passed to a FSC-W/FSC-H gate to exclude FSC-doublet cells. Third, cells were passed to a SSC-W/SSC-H gate to exclude SSC-doublet cells. Cells were then gated on Viability/CD4. Viability- CD4+ cells were passed on and used to determine individual's memory (CCR7/CD45RA) and chemokine (CXCR3/CCR4/CCR6) expression, which was used later when applied to individual person's tetramer+ cells.

Higher order positives were removed from each tetramer-channel by plotting corresponding other tetramer channels vs each other and excluding all higher order positives. Once higher order positives were removed, those tetramer+ cells were used for final analysis.

For human beta cells:
Pancreatic islet cells were first gated on SSC-A/FSC-H. Second, cells were gated on Zinc+ (ie FITC channel) cells. Third, cells were passed to a FSC-W/FSC-H and subsequently SSC-W/SSC-H to exclude doublets. Cells were then collected by zinc+ and TMRE+ cells to enrich viable beta cells.

☒ Tick this box to confirm that a figure exemplifying the gating strategy is provided in the Supplementary Information.
